# Prenatal diagnosis of fetal defects and its implications on the delivery mode

**DOI:** 10.1515/med-2023-0704

**Published:** 2023-05-12

**Authors:** Pawel Sadlecki, Malgorzata Walentowicz-Sadlecka

**Affiliations:** Department of Obstetrics and Gynecology, Regional Polyclinical Hospital Grudziadz, Poland

**Keywords:** congenital defects, congenital malformations, fetal defects, delivery mode, cesarean delivery, vaginal delivery

## Abstract

Congenital malformations are defined as single or multiple defects of the morphogenesis of organs or body parts, identifiable during intrauterine life or at birth. With recent advances in prenatal detection of congenital malformations, many of these disorders can be identified early on a routine fetal ultrasound. The aim of the present systematic review is to systematize the current knowledge about the mode of delivery in pregnancies complicated by fetal anomalies. The databases Medline and Ebsco were searched from 2002 to 2022. The inclusion criteria were prenatally diagnosed fetal malformation, singleton pregnancy, and known delivery mode. After the first round of research, 546 studies were found. For further analysis, studies with full text available concerning human single pregnancy with known neonatal outcomes were considered. Publications were divided into six groups: congenital heart defects, neural tube defects, gastroschisis, fetal tumors, microcephaly, and lung and thorax malformations. Eighteen articles with a descripted delivery mode and neonatal outcome were chosen for further analysis. In most pregnancies complicated by the presence of fetal anomalies, spontaneous vaginal delivery should be a primary option, as it is associated with lower maternal morbidity and mortality. Cesarean delivery is generally indicated if a fetal anomaly is associated with the risk of dystocia, bleeding, or disruption of a protective sac; examples of such anomalies include giant omphaloceles, severe hydrocephalus, and large myelomeningocele and teratomas. Fetal anatomy ultrasound should be carried out early, leaving enough time to familiarize parents with all available options, including pregnancy termination, if an anomaly is detected.

## 1 Introduction

Congenital malformations are defined as single or multiple defects of the morphogenesis of organs or body parts, identifiable during intrauterine life or at birth. Anomalies, either major or minor (including malformations, deformations, disruptions, dysplasia, and sequences), can occur as isolated phenomena or as component manifestations of broader patterns or syndromes and are causally heterogeneous [1]. Congenital defects may be induced by genetic or environmental factors or combinations thereof in a multifactorial etiology. Congenital anomalies have four etiologies: malformation, disruption, deformation, and dysplasia [1]. The global prevalence of fetal malformations at birth is estimated to be 2–3% [2].

The availability of new genetic testing techniques and continuous progress in fetal imaging technologies have fundamentally changed the practice of prenatal diagnosis during the past decades [3]. Recent advances in prenatal diagnosis give us the opportunity to identify pregnant women who are at high risk of developing conditions such as preeclampsia or fetal growth restriction [4,5]. On the other hand, cell-free fetal DNA (cffDNA) analysis is a valuable tool in prenatal diagnosis, providing a safe and reliable means of screening for fetal pathologies. cffDNA analysis is a non-invasive prenatal diagnostic test with the fundamental role of screening chromosomic or monogenic pathologies of the fetus. The test can be administered for fetal DNA detection in the mother’s blood from the fourth week of gestation [6].

With recent advances in prenatal detection of congenital malformations, many of these disorders can be identified early on a routine fetal ultrasound. Whenever it occurs, a pregnant woman should consult an experienced team, including a perinatology specialist and pediatric surgeon, as soon as possible to ensure that specialized personnel and resources will be available in the delivery room to provide appropriate intervention to the neonate with a potentially life-threatening malformation. A crucial step in prevention strategies to reduce the rate of stillbirth is the identification of major possible causes of stillbirth. The second-trimester uterine arteries’ pulsatility index (PI) is a valuable tool in prenatal diagnosis that provides insights into stillbirth etiology. This measurement is a function of placental pathology and can be used to detect potential issues with the placenta that could lead to stillbirth [7].

In recent years, a centralization of perinatology centers involved in diagnosing, treating, and providing safe delivery in pregnancies complicated with fetal malformations can be observed in Europe [8]. Such centralized centers offer highly specialized invasive procedures that can save the fetus’s life, change the natural course of the disease, or improve the overall condition of the neonate after birth [9]. Depending on the type of anomaly present and experience of the team, treatment may be through open surgery hysterotomy or minimally invasive surgical techniques through fetoscopy. Fetal surgical intervention can be a reasonable option in some selected congenital anomalies, such as large congenital cystic adenomatoid malformations (CCAMs) with the signs of hydrops, giant sacrococcygeal teratomas, severe congenital diaphragmatic hernias (CDHs), and meningomyelocele [10]. Congenital anomalies and major chromosome abnormalities pose a higher risk of malpresentation, which may protract labor and increase the risk of cesarean delivery. Many authors found a link between the presence of fetal anomalies and certain obstetrical conditions, such as malpresentation, which leads to increased cesarean delivery rates. Other reasons behind the increasing frequency of cesarean deliveries in pregnancies with prenatally diagnosed fetal anomalies include the lack of clear-cut recommendations from scientific bodies about the mode of delivery and physicians’ concerns about legal liability in the case of vaginal delivery failure. The aim of the present systematic review is to systematize the current knowledge about the mode of delivery in pregnancies complicated by fetal anomalies.

## 2 Methods

**Figure 1:**
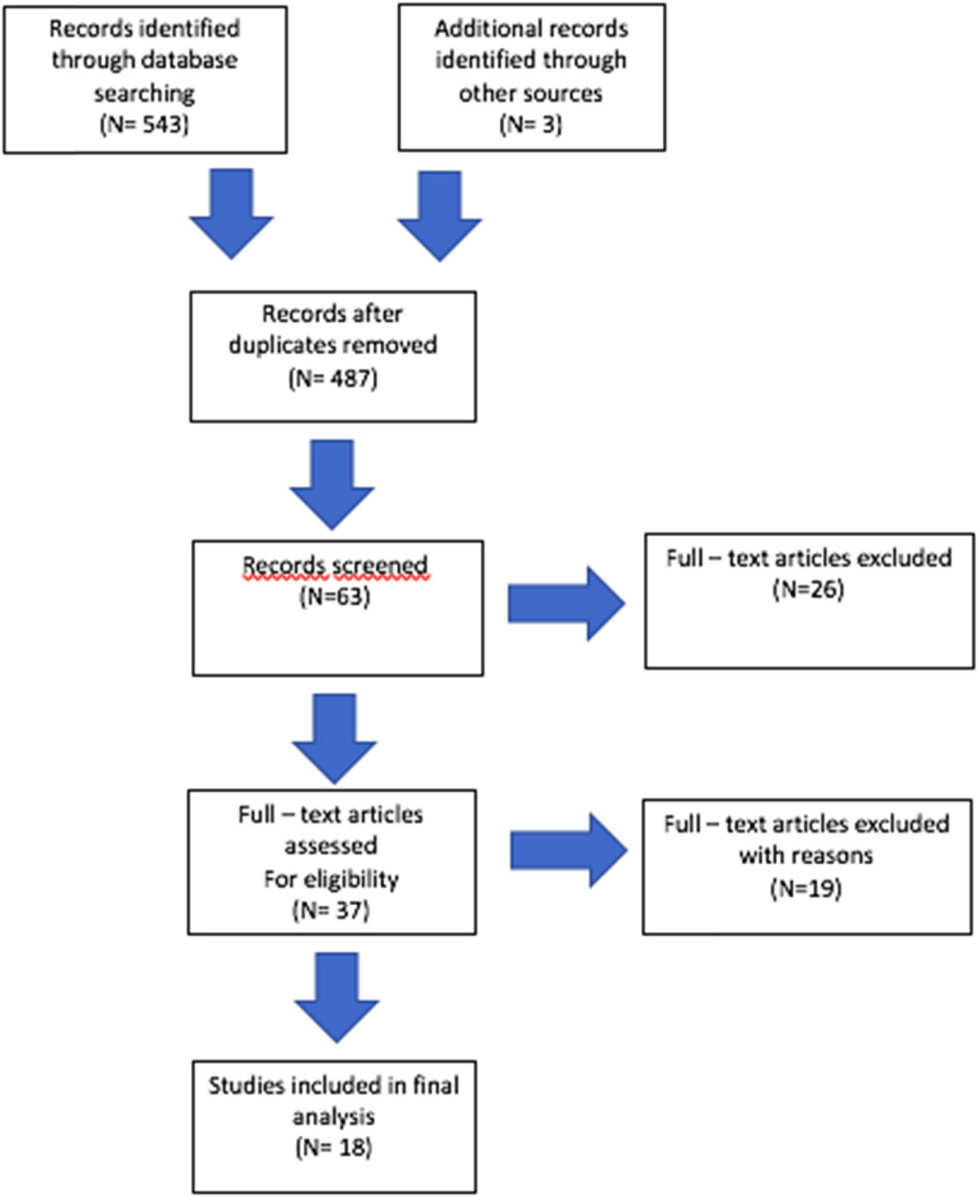
A flow diagram of the study selection process.

The following systematic review was carried out in line with the international standards and guidelines for systematic reviews (PRISMA). Detailed review protocol can be obtained from the author upon request. The databases Medline and Ebsco were searched from 2002 to 2022. The inclusion criteria were prenatally diagnosed fetal malformation, singleton pregnancy, and known delivery mode. The exclusion criteria were obstetrical indications for cesarean delivery, chromosomal abnormalities, fetal demise, multiple pregnancies, and lack of neonatal outcomes. Eligible studies were found using a combination of the following key words: congenital defects, congenital malformations, fetal defects, delivery mode, cesarean delivery, and vaginal delivery. The language was limited to English; duplicated articles and studies without full text available were excluded from further study. Peer-reviewed observational studies and retrospective analysis were considered. The results were restricted to English-language publications concerning the most frequent fetal malformations: congenital heart defects (CHDs), neural tube defects (NTDs), gastroschisis, fetal tumors, microcephaly, and lung and thorax malformations. The Newcastle-Ottawa Scale was implemented to assess the quality of the included studies. A secondary search included examining the reference lists of all included articles; we also used Google Scholar to complete our inquiry of recently published studies. After careful consideration, we have excluded case reports, abstracts, validation, and animal studies. A flow diagram of the study selection process is presented in Figure 1.

## 3 Results

After the first round of research, 546 studies were found. Publications were limited to the six most common groups: CHDs, NTDs, gastroschisis, fetal tumors, microcephaly, and lung and thorax malformations. For further analysis, studies with full text available concerning human single pregnancy with known neonatal outcome were considered. A secondary search allowed for choosing 63 publications, and studies with number of participants below 20 were excluded. Then, among 37 articles, we found 18 that met the criteria with a descripted delivery mode and neonatal outcome. Selected publications were considered for further analysis. The analyzed publications are presented in Table 1.

**Table 1:** Publications analyzed in the review

Reference	Type of study	*N*	Fetal malformation	Vaginal delivery (VD)	Cesarean delivery (CD)	Outcome
Asoglu et al. [17]	Retrospective cohort study	222	CHD	Attempted VD 177 (79.8%)	Elective CD 45 (20.2%)	UA pH < 7.1 4(8.8), elective CD (*N* = 45)
						UA pH < 7.1 10(5.6) attempted VD (*N* = 177)
				Successful VD 142 (80.2%)		UA pH < 7.1 3(2.1) successful VD (*N* = 142)
					CD after attempted VD 35 (19.8%)	
						UA pH < 7.1 7(20.0) intrapartum CD (*N* = 35)
Zloto et al. [19]	Retrospective cohort study	105	CHD	VD 76 (72.3%)	CD after attempted VD 10 (9.5%)	The operative VD rate was significantly higher in the CHD
	(the healthcare database of a tertiary medical center)			Assisted VD 19 (18.1%)		
						No difference between the CHD and the control group in the rate of intrapartum CD
						The most common indication for operative VD was the non‐reassuring fetal heart rate
Ge et al. [14]	Retrospective cohort study	170 CHD	CHD		CD 42.9% (CHD)	40% of the CHD and 36% of the non-cardiac anomaly group had 1-minute Apgar scores of < 8 compared to 26% in the non-anomalous group (*p* < 0.001)
		234 non-cardiac anomaly				
					CD 44.9% (other anomalies)	
						5 min Apgar scores of < 8 were 24% of CHD, 18% of other anomalies, and 10% of the control group (*p* < 0.001)
					CD 35.1% (controls)	
						CHD was associated with lower umbilical artery cord pH (7.2 versus 7.3, *p* = 0.013)
						No significant difference between base excess (−3.5 versus −3, *p* = 0.117)
Weissmann-Brenner et al. [15]	Retrospective cohort study	1598	CHD	Spontaneous VD 1010 (63.2%)	CD 507 (31.7%)	5 min Apgar < 7
		688, 43.1% were diagnosed prenatally				CHD 22 (1.4%)
					Elective CD 266 (16.6%)	
				Operative VD. 81 (5.0%)		Controls 261 (0.3%)
					Elective CD for maternal request 18 (1.1%)	
Al-Obaidly et al. [24]	Retrospective cohort study	42	NTD	VD 69% (29/42)	CD 31% (13/42)	—
					Primary CD 31% (4/13) Repeat CD 69% (9/13)	
		singleton infants with isolated anencephaly after 24 weeks				
Farmer et al. [32]	Retrospective cohort study	91 prenatally diagnosed	NTD	—	CD 91 (100%) after open fetal surgery	—
		Open-hysterotomy repair	Myelomeningocele			
Goodnight et al. [33]	Prospective observational registry Fetal Myelomeningocele Consortium	52 prenatally diagnosed pregnancies progressing > 20 weeks	NTD	—	100% after open fetal surgery	96.2% live birth rate
			Open NTD			
		Open-hysterotomy repair				
King et al. [34]	Retrospective cohort study patients who underwent prenatal Open Neural Tube Defect repair open vs fetoscopic	78	NTD	Spontaneous VD 4/22 (64%)	Scheduled CD 9/25 (36%)	In the fetoscopic-repair group, compared with the open-repair group, the median gestational age at birth was significantly higher (38.1 weeks (IQR, 35.2 –39.1 weeks) vs 35.7 weeks (IQR, 33.9–37.0 weeks); adjusted *p* < 0.001) and about twice as many infants were born at term (68% vs 35%; adjusted *p* = 0.027). No significant differences in other maternal or neonatal outcomes between the two groups.
		47 fetoscopic				
		31 open hysterotomy			Elective CD 11/25 (44%)	
					Emergency CD 5/25 (20%)	
Nitzsche et al. [36]	Retrospective cohort study	33	Gastroschisis	—	CD 33 (100%)	All fetuses were born alive
Overcash et al. [37]	Retrospective cohort study	191	Gastroschisis	VD 126 (66%)	CD 65 (34%)	No differences in outcomes with vaginal or cesarean delivery.
						75 (39%) deliveries were elective. Those electively delivered had a later gestational age at delivery compared to those not electively delivered (37 0/7 ± 1.4 weeks compared with 36 0/7 ± 1.9 weeks, *p* < 0.0001)
						Infants electively delivered had higher birth weights (2,600 ± 472 g compared with 2,384 ± 500 g, *p* < 0.01) and lower rates of preterm delivery (40% compared with 73%, *p* < 0.0001)
Martins et al. [41]	Prospective case-series study	79	Gastroschisis	VD 26 (33%)	CD 53 (67%)	The available data do not support a policy of cesarean delivery for infants with gastroschisis, and it should be reserved for the usual obstetrical indications.
						A significantly higher risk of morbidity and mortality among complex patients than among simple patients
						A few advantages of the sutureless technique over the sutured technique for closing the abdominal defect
Palatnik et al. [43]	Retrospective cohort study	206	Gastroschisis	VD early preterm (31 + 0 to 34 + 6 weeks) 17 (56.7)	CD early preterm (31 + 0 to 34 + 6 weeks) 19 (43.2)	The CD was more frequent during the early and late preterm groups than during the term group
				VD late preterm (35 + 0 to 36 + 6 weeks) 72 (77.4)	CD late preterm (35 + 0 to 36 + 6 weeks) 32 (22.9)	
				VD term (≥ 37 + 0 to 39 + 6 weeks) 3 (15.0)	CD term ( ≥ 37 + 0 to 39 + 6 weeks) 14 (82.4)	
Kamil et al. [46]	Retrospective cohort study	*84*	Fetal tumors	VD 36.2%	CD 63.8%	69/84 (82.1%) fetuses were liveborn. In 12 cases, the pregnancy was terminated; 3 cases of intrauterine death
Wilson et al. [56]	Retrospective cohort study	*26*	Fetal tumors Sacrococcygeal teratoma	VD 2	CD (low transverse) 10	In the 3 of the 5 cases with perinatal mortality, 1 of intrauterine fetal death at 29 weeks, 2 neonatal deaths with extreme prematurity at 25, and at 30 weeks and 5 days.
					CD (classical) 4	
Bardin et al. [60]	Retrospective cohort study	3677	Microcephaly	VD spontaneous 2993 (82.0%)	CD intrapartum 232 (6.3%)	Fetuses with isolated microcephaly have a similar mode of delivery and perinatal outcome to fetuses with a normal HC
				VD operative 426 (11.7%)		
Soni et al. [69]	Retrospective cohort study	185	CDH	VD early term (37–38.6) spontaneous 25; induced 33	CD early term (37–38.6)	No difference between early term and full term
					Unplanned 17; elective 38	
				VD full term (39–40.6)		
					CD full term (39–40.6)	
				Spontaneous 6; induced 31	Unplanned 22; elective 13	But the trend to lower ECMO and improved survival
Bouchghoul et al. [67]	Retrospective cohort study	213	CDH	VD 140	CD 70	Survival after 28 days did not differ according to the mode of delivery. (But the trend to lower survival for babies born by CD vs VD.)

Burgos et al. [70]	Retrospective analysis of data CDH Study Group	3906 prenatally diagnosed	CDH	VD spontaneous 1119 29%	CD elective 1107 28%	Overall survival
				VD induced 747 19%	CD emergent 805 21%	VD spontaneous 30%
						VD induced 20%
						CD elective 29%
						CD emergent 21%

*p*: probability value, *N*: number of participants, CDH: congenital diaphragmatic hernia, CHD: congenital heart defect, and NTD: neural tube defect.

## 4 Discussion

### 4.1 Fetal CHDs

CHDs are the most common fetal structural anomalies, with the incidence ranging between 8 and 12 per 1,000 live births depending on the time of diagnosis [11]. The incidence of CHDs in early gestation can be even higher, given that some CHDs are complex and usually lead to fetal demise [12]. A growing number of CHDs are diagnosed *in utero* with improvements in ultrasonographic technology and an increase in the demand for prenatal screening. Up to 50–60% of CHDs require surgical corrections, and 25% of conditions from this group are critical, constituting a leading cause of infant mortality [12]. Due to prompt detection of a severe CHD that requires intervention shortly after delivery, planned intervention can take place at a tertiary center with a congenital cardiac surgery program implemented and units equipped to treat such cases, or at a hospital located within the proximity of such a center. Pregnancies with prenatally and postnatally diagnosed severe CHDs requiring neonatal surgical intervention differed significantly in terms of perinatal management and delivery planning [13]. Although the complexity of CHDs found in the prenatally and postnatally diagnosed groups was similar, neonates from the former group were born earlier and with lower birth weights than those diagnosed postnatally [13]. With the prenatal diagnosis, there is an opportunity to schedule the delivery at a tertiary center capable of offering a necessary cardiac intervention following a prompt evaluation by a multidisciplinary team of cardiology, surgery, intensive care, and neonatology specialists. Thus, the delivery location for pregnant women with a prenatal diagnosis of a CHD posing the risk of postnatal compromise should be planned appropriately. It was proved that prenatal diagnosis of CHDs was associated with lower birth weight, preterm delivery, and increased risk of primary cesarean delivery [14].

There is evidence that pregnancies with fetal CHD were significantly more often delivered by cesarean section due to non-reassuring fetal heart rate regardless of prenatal diagnosis of the anomaly [15]. Moreover, cases of neonates with CHDs have a greater tendency toward an unfavorable outcome (e.g., 5 min Apgar score < 7, seizures, and hypoxic-ischemic encephalopathy) independently of prenatal diagnosis of CHDs [15]. Additionally, according to the published evidence, the overall neonatal conditions and surgical outcome are better when the delivery occurs in close proximity of a cardiac center capable of providing surgical interventions for neonates with major CHDs [16]. However, there is still no consensus considering the optimal mode of delivery in pregnancies with fetal CHDs. The risk of maternal and fetal comorbidities was increased, regardless the type of cesarean delivery: elective or intrapartum. The severity of CHDs did not seem to change either the delivery route or immediate neonatal outcomes; the paramount importance in determining the mode of delivery in pregnancies with fetal CHDs was identification of preexisting obstetrical comorbidities [17]. Vaginal delivery was successful in 66.1% of cases of fetal cardiac anomalies [11]. Planned cesarean delivery in pregnancies with prenatally diagnosed fetal cardiac anomalies was more common than in pregnancies in which such anomalies were detected postnatally. Importantly, the planned cesarean delivery did not reduce the risk of composite neonatal morbidity compared with attempted vaginal delivery [11].

Neonatal outcomes of fetuses with CHDs can be improved by delaying elective delivery to at least 39 weeks; however, waiting beyond 42 weeks turned out to have a negative impact on their condition [18]. These findings are in contrast to the results of some recent studies that identified a small albeit significant negative trend in gestational age at delivery in infants diagnosed prenatally with single-ventricle defects [16]. After excluding elective cesarean deliveries, operative vaginal deliveries due to non-reassuring fetal heart rates were more common among CHD patients [19]. Moreover, neonates diagnosed prenatally with CHD were more often small for gestational age, had meconium-stained amniotic fluid, 1 and 5 min Apgar scores ≤ 7, non-reassuring fetal heart rate, and chorioamnionitis [19]. Physicians should be aware of these findings and consider them when deciding on the mode of delivery. Also, good communication between obstetrical and cardiological units is essential in this setting because elective labor induction before 39 weeks of gestation is not recommended for fetuses with CHDs unless patient-specific obstetrical or logistic issues or fetus-specific concerns about well-being exist [13]. The decision to terminate pregnancy in cases of severe anomalies or to refrain from intervention in favor of palliative care at birth is a complex and highly individualized process. Complex counseling should provide parents with all necessary support regardless of their choice. They should refrain from presenting their personal opinions during the discussion and focus on providing families with all the support essential to arrive at a decision. Informed consent should be driven by evidence and patient’s education. The purpose of informed consent is to enable the patient to make an informed decision.

### 4.2 NTDs

NTDs are complex and heterogenous groups of the congenital central nervous system (CNS) anomalies resulting from the failure of the neural tube to close [20]. This group includes several anomalies, e.g., anencephaly, spina bifida, and encephalocele. The incidence of NTDs has been reported at 0.89–0.93 per 1,000 live births in Europe, 0.53 per 1,000 in the United States, 0.2–9.6 per 1,000 in Latin America, and 0.62–13.8 per 1,000 in Arab countries [21]. Risk factors for NTDs include maternal folate deficiency, obesity, diabetes mellitus, fungus contaminants of maize, mycotoxins, heat, exposure to teratogens (valproic acid, carbamazepine, lead), influenza virus, and socioeconomic status [22].

Anencephaly is a severe pathology of neuroaxis development arising 26–28 days after conception due to impaired closing of the neural tube developing forebrain and variable amounts of brainstem being fully exposed [23]. Although some fetuses are born alive, anencephaly is recognized as a lethal condition with no possibility of effective treatment [24]. The global prevalence of anencephaly is estimated to be 5.1 per 10 000 births; in addition in countries where termination of pregnancy is prohibited, the incidence of anencephaly tends to be even higher [25,26]. Prenatal detection of anencephaly is possible during the first trimester ultrasound scan and it is considered reliable, and less than 25% of anencephaly is detected by ultrasound prior to 18 weeks of gestation [27]. During the first trimester, an early fetal anatomy assessment can detect not only structural non-genetic malformation but also anomalies associated with genetic defects different from the common chromosomal aberrations [28]. According to many authors, pregnancy with anencephaly should be terminated via the vaginal route, although induced labor may increase the risk of cesarean delivery [29]. There is also some evidence that compared with labor of spontaneous onset, elective labor induction in nulliparous women is associated with significantly more operative deliveries. Moreover, the difference in the delivery mode could be attributed to the gestation age and the risk of cervical damage by the bony basal skull of the fetus [29]. Additionally, the incidence of shoulder dystocia may increase due to a smaller head circumference (HC) in anencephalic fetuses, which enables full dilatation of the cervix and increases the risk of the shoulders getting stuck [30]. Another important problem is dysfunction of the hypothalamic-pituitary axis in anencephalic pregnancies, which leads to prolonged gestation that increases the risk of repeated cesarean delivery in women with a previous uterine scar [31].

Another example of NTDs is myelomeningocele. A large body of evidence shows that this anomaly can be successfully treated surgically during the prenatal period. The Management of Myelomeningocele Study demonstrated that morbidity associated with spina bifida can be reduced by fetal myelomeningocele closure via open maternal-fetal surgery. The documented beneficial effects of the treatment included the reduced need for ventriculoperitoneal shunting in the first year of life, reversal of hindbrain herniation, and improvement of motor function and neurodevelopmental outcomes at 30 months of age [32]. Open maternal-fetal surgery is performed via hysterotomy in the contractile portion of the uterus, similar to that created during a classical cesarean delivery. Since conventional hysterotomy is associated with a 4–9% risk of uterine rupture and a resultant increase in maternal and neonatal morbidity, the risk in pregnancy following open surgery can be similar or even greater [33]. A number of recently conducted studies demonstrated the effectiveness and safety of endoscopic treatments for NTDs. Compared with an open approach, fetoscopic repair of open NTDs was shown to reduce the incidence of cesarean delivery, preterm delivery, and other important complications, such as uterine scar dehiscence, with no significant difference in total costs of care from surgery to infant discharge [34]. During preoperative counseling, patients considering maternal-fetal surgery for myelomeningocele closure should be informed not only about the benefits in terms of the reduction of morbidity for the neonate and child but also about the associated maternal risks for the index pregnancy, as well as about the potential additive risk of uterine scar complications in future pregnancies. All those risks should also be considered during the management of subsequent pregnancy; thus, women with a history of maternal-fetal surgery for myelomeningocele closure in previous pregnancies should optimally be offered delivery at 36–37 weeks of gestation and be managed in close collaboration with a center having adequate expertise in fetal surgery [33]. Moreover, the decision on the mode of delivery should be based on prenatal diagnosis and the type of fetal therapy. If both open and endoscopic approaches are available at the perinatal center, both should be discussed because the later decision on the mode of delivery is also based on the fetal therapy used previously [34]. In the case of large anomalies with concomitant hydrocephalus, cesarean delivery is a highly recommended option. However, in the case of fetuses whose prognosis is grave based on the lesion level and size or *in utero* movement, vaginal delivery may be a preferable option. The results of a large meta-analysis suggest that routine cesarean delivery for fetuses with open NTDs does not necessarily improve neurological outcomes; however, it needs to be emphasized that available evidence on this matter is limited to small observational studies [35].

### 4.3 Gastroschisis

Gastroschisis is an abnormality of the abdominal wall that results in the herniation of the bowel and other abdominal organs, typically on the right side of normal umbilical cord insertion. Thanks to the progress in ultrasound technology, the majority of gastroschisis cases, approximately 90–98%, can be identified prenatally nowadays. The prevalence of gastroschisis has been estimated to be 0.5–1 per 10,000 births [36]. However, recent evidence suggests that the prevalence of gastroschisis is increasing, up to 5.1 per 10,000 births [36]. While the overall fetal-neonatal mortality due to gastroschisis remains relatively low (5–10%), pregnancies complicated by this anomaly are often associated with fetal growth restriction, preterm delivery, and neonatal complications, such as bowel atresia, perforation, stricture, ischemia, and necrotizing enterocolitis [37]. Risk factors for gastroschisis include young maternal age, tobacco and illicit drug use, low socioeconomic status, low body mass index, and infections [38]. The intestinal wall undergoes changes with the severity of inflammation, as shown histologically in both human studies and experimental animal models. The inflammatory changes may be mediated by exposure to amniotic fluid constituents. In 17% of the fetuses with gastroschisis, the exposed bowel coexists with other defects, such as intestinal atresia, necrosis, perforation, or volvulus, which is referred to as complex gastroschisis. The risk of morbidity and mortality in such patients is higher than in neonates with simple gastroschisis without concomitant intestinal defects [39]. The optimal timing and mode of delivery in pregnancies with gastroschisis are still a matter of debate. Some authors recommend elective preterm delivery to minimize the exposure of the gastrointestinal tract to the proinflammatory constituents of the amniotic fluid. Published evidence suggests that the induction of labor at 37 weeks of gestation was associated with lower rates of sepsis, bowel damage, and neonatal death compared with pregnancies managed expectantly beyond 37 weeks [40]. However, no difference in the outcomes of pregnancies terminated by elective delivery at 36 weeks and those treated expectantly until spontaneous labor was demonstrated in one randomized controlled trial [37]. Also, an optimal delivery mode in pregnancies complicated by gastroschisis raises some controversies. According to the literature, gastroschisis does not constitute an indication for routine cesarean delivery, and the decision on the mode of delivery should be based primarily on obstetrical conditions and the clinician’s and parents’ discretion [41,42]. There are centers that do not perform cesarean deliveries routinely in pregnancies with gastroschisis managed at their center, and the time of delivery for neonates with this anomaly is not arbitrarily controlled. However, as stated by some authors, neonates with gastroschisis delivered during nighttime received delayed closure more frequently [43]. Thus, scheduling the delivery in the morning hours, when all necessary specialists are available at the clinic, seems to be a reasonable option. According to many authors, the outcomes of vaginal deliveries and cesarean deliveries in pregnancies with gastroschisis are essentially the same; hence, this anomaly should not be considered an indication for elective cesarean delivery. Considering that cesarean delivery is known to be associated with increased maternal morbidity and lack of neonatal benefit, scheduled cesarean delivery in pregnancies with gastroschisis should not be recommended.

### 4.4 Fetal tumors

The estimated prevalence of all congenital tumors varies between 1 and 13.5 per 100,000 live births, and the true number of these anomalies may be higher as such conditions are likely not reported [44]. Detection of a fetal tumor constitutes a diagnostic and therapeutic dilemma given the variety of possible differential diagnoses and the variable course during the pregnancy and after birth [45]. The most challenging problem faced by a physician is the decision of whether a given case is eligible for ultrasonographic monitoring with a surgical intervention postponed postnatally. Frequently, it is impossible to distinguish between the fetal masses that require a prenatal intervention and those cases where immediate postnatal treatment is also an option [46]. Many fetal tumors are often found incidentally during the second- or third-trimester ultrasound examination [47].

It is estimated that fetal intracranial tumors account for 0.5–1.9% of all tumors detected in children [48]. Teratoma is the most commonly occurring brain tumor during fetal development, while astrocytoma, craniopharyngioma, and primitive neuroectodermal tumor are less common [48]. These tumors are typically detected during the third-trimester ultrasound scan. Unfortunately, fetal intracranial tumors often have a poor prognosis and can cause complications such as intracranial bleeding or dystocia during delivery [49]. The most common life-threatening fetal masses of the head and neck include vascular lesions, such as lymphangioma and hemangioma; these two types of lesions are often considered congenital malformations rather than true neoplasms [50].

Teratomas are neoplasms containing the derivatives of all three germ layers, usually, they are benign and very seldom undergo malignant transformation. Oropharyngeal teratoma, also referred to as an epignathus, constitutes 2–3% of all congenital teratomas. Cervical teratomas are usually detected as large masses that may extend in all directions. They may contribute to a significant hyperextension of the fetal neck, which may result in dystocia. The prognosis in fetal teratoma depends on the size and location of the mass and the consequent risk of airway obstruction [51]. Lymphatic malformations of the fetal head and neck are benign lesions very often found as fluid-filled, multiseptate cysts. They typically arise in the anterior or posterior triangles of the neck, and sometimes if venous components are present, they contain *phleboliths* – focal calcifications. Lymphatic malformations may be the cause of a lethal airway obstruction that requires intubation promptly after delivery and may lead to infection if left untreated. Optimal treatment of prenatally diagnosed teratomas and lymphatic malformations typically involves surgical excision, and the prognosis after treatment is usually very good [52].

The incidence of sacrococcygeal teratoma is estimated to be 1 in 40,000 live births, and it is more commonly diagnosed in female infants [53]. Typically, sacrococcygeal teratomas are present as an exophytic mass located in the sacrococcygeal area. Depending on the location of the mass relative to the body, four types are distinguished: type I is external, type II is equally located both externally and internally, type III is mainly internal, and type IV is entirely internal [54]. The presence of a sacrococcygeal teratoma may increase perinatal morbidity and mortality due to dystocia, fetal hydrops, polyhydramnios, bleeding, or cardiovascular complications [55]. Prognosis related to vascular complications caused by the tumor can be determined based on the serial evaluation of the heart size and Doppler measures of the fetal cardiac output [56]. Doppler interrogation of the umbilical arterial flow showing diminished or reversed diastolic flow reflecting competitive “steal” from the placenta to the sacrococcygeal teratoma is a marker of poor outcome [57]. Mirror syndrome is a life-threatening condition associated with maternal fluid retention and hemodilution; the syndrome occurs concomitantly to fetal hydrops and manifests as progressive maternal edema “mirroring” that of the affected fetus [58]. The treatment of sacrococcygeal teratomas involves surgical removal of the tumor mass using either open surgery or laparoscopic closure of the vessels supplying the tumor. The delivery method for fetuses with this condition depends on several factors, including the size of the tumor and the fetal condition. Vaginal delivery may still be an option for fetuses with an uncomplicated tumor < 5 cm in diameter; otherwise, cesarean delivery is the preferred method [59].

### 4.5 Microcephaly

Microcephaly, which is defined as an HC that is smaller than 3 SD below the mean for a person’s age and sex, is a neurological marker, which can be detected antenatally during a routine ultrasonographic examination or post-partum during the neonatal physical examination. Microcephaly is a relatively rare condition, with an estimated prevalence of 0.1–0.5% [60]. The diagnosis of microcephaly can be challenging, given a plethora of available cut-off values. The prognosis varies depending on the severity of microcephaly, its etiology, and accompanying manifestations [60]. HC below 3 SD of the mean is more likely to be associated with various disorders, whether genetic or non-genetic, that affect the development of the brain and contribute to intellectual disability and neurological abnormalities [60]. Abnormal pelvic proportions or fetal head dimensions that are too small or too large can lead to prolonged or arrested labor and may increase the risk of instrumental delivery and maternal and fetal complications [61]. Increased biparietal diameter in a term fetus at up to 7 days before labor was found to be associated with a significantly higher rate of operative vaginal deliveries without adverse neonatal outcomes [60]. Large HC was shown to be an independent risk factor for operative vaginal delivery and unplanned cesarean delivery; maternal and fetal issues should be considered, including primiparity or multiparity [62]. The mode of delivery and perinatal outcomes in fetuses with isolated microcephaly are similar to those in those with a normal HC. Further prospective studies based on valuable pre-labor and ultrasonographic data are needed to understand better the effect of microcephaly on labor progress and perinatal factors.

### 4.6 Lung and thorax malformations

CCAM of the lung is a rare defect of the terminal bronchioles. CCAM occurs due to an alteration in embryogenesis between the fifth and seventh week of gestation [63]. The overall prevalence of CCAM remains unknown, partly due to the non-universal nature of prenatal ultrasound screening and partly because of the variety of diagnostic classifications and criteria. The reported prevalence varies between 1:25,000 and 1:35,000 live births [64]. Prenatal diagnosis can be established with ultrasonographic techniques. If a fetal thoracic mass is detected in ultrasound, CCAM should be considered as a differential diagnosis, along with CDH, bronchopulmonary sequestration, and other, much less frequently observed, anomalies such as bronchogenic or enteric cysts, neuroblastoma, cerebral heterotopia, congenital lobar emphysema, mediastinal cystic hygroma, and unilateral bronchial atresia [64]. Macrocystic CCAM can mimic CDH with herniated stomach and intestines. Bronchopulmonary sequestration, with an ultrasonographic presentation of a well-defined homogenous mass, is characterized by the presence of an aberrant nutrient artery, absent in the case of CCAM. The prognosis varies with some anomalies undergoing spontaneous intrauterine resolution without observable pathologies found after birth. The large CCAM can interfere with the cardiovascular function of the fetus, changing loading conditions due to cardiac compression and the creation of tamponade-like physiology. Progressive changes, corresponding to alterations in ventricular filling and compliance, can be identified on serial fetal echocardiography with Doppler interrogation. In some cases, those abnormalities may lead to mortality due to pulmonary hypoplasia or moderate respiratory symptoms during childhood, and some persistent lesions can cause recurrent infections and even undergo malignant transformation [65]. Thus, surgery is usually necessary after birth. A large CCAM with early signs of hydrops is a fatal condition, and fetal surgical intervention should be considered as rescue therapy in such cases.

CDH is a congenital birth defect that occurs in approximately 1 in 3,000 live births [66]. The main determinants of short-term prognosis in such cases are persistent pulmonary hypertension of the newborn and the degree of pulmonary hypoplasia. Preterm delivery in CDH fetuses is associated with poor outcomes, with survival rates of 35–43% for newborns delivered before 37 + 0 weeks gestation [67]. CDH can be managed prenatally through the laparoscopic percutaneous endoscopic tracheal occlusion. Deployment of an occlusive balloon within the fetal trachea may promote lung growth and improve neonatal outcomes [68]. Unfortunately, such therapy is associated with an increased rate of preterm delivery. Published evidence shows that the mode of delivery in pregnancies with CDH (vaginal delivery vs cesarean delivery) is not associated with neonatal morbidity or mortality [68,69]. The survival rates in the case of planned cesarean delivery and vaginal delivery were shown to be similar; a lower survival rate was documented solely in the subgroup in which emergency cesarean delivery was conducted after an unsuccessful attempt at vaginal delivery [70]. Based on these findings, the mode of delivery in pregnancies with CHD should be chosen primarily based on obstetrical indications, as this anomaly alone does not constitute a sufficient reason for planned cesarean delivery. The tracheal balloon should be removed by *ex utero* intrapartum treatment (EXIT). The EXIT procedure is an established method to secure obstructed neonatal airways during delivery while still maintaining fetoplacental circulation for 20–30 min or more [71]. To be performed successfully, the EXIT procedure requires coordination and collaboration among physicians of maternal-fetal medicine, neonatological intensive care, obstetrical and pediatric anesthesiology, interventional radiology, and pediatric otolaryngology–head and neck surgery, as well as two teams of surgical nurses.

## 5 Conclusions

In most pregnancies complicated by the presence of fetal anomalies, spontaneous vaginal delivery should be a primary option, as it is associated with lower maternal morbidity and mortality. Given the risks related to prematurity, preterm delivery should be recommended solely in rare, selected cases, with the most likely indication being declining fetal status. In some cases, such as progressive heart failure or significant progression of the anomaly, interfering with the normal function of body organs or systems, preterm delivery may be necessary to optimize fetal outcomes. Planned labor induction may be beneficial for women who reside far away from a tertiary care center. Cesarean delivery is generally indicated if a fetal anomaly is associated with the risk of dystocia, bleeding, or disruption of a protective sac; examples of such anomalies include giant omphaloceles, severe hydrocephalus, large myelomeningocele, and teratomas. The EXIT procedure may be offered in the case of complex airway masses or CDH but it needs to be stressed that the procedure is associated with much higher maternal risk than a cesarean delivery. Fetal anatomy ultrasound should be carried out early, leaving enough time to familiarize parents with all available options, including pregnancy termination, if an anomaly is detected.

The optimal approach in the case of prenatal detection of a fetal anomaly is to provide the pregnant woman with comprehensive specialized care. A perinatologist/maternal-fetal medicine specialist confirms the anomaly and determines the prognosis and possibilities for intrauterine treatment. The obstetrician, in agreement with the neonatologist, suggests the optimal mode of delivery and peri-delivery management, while the pediatric surgeon presents the options for surgical treatment and correction of the anomaly. A team of specialists determines the frequency of follow-up visits, the timing, and the facility where the delivery should take place. The couple has the opportunity to receive psychological support during the pregnancy and after delivery. The choice of the delivery mode depends on the type of anomaly, the type of treatment performed, and the need for specialized procedures after the child’s birth. The suggested management of pregnancies complicated with fetal anomalies is presented in Table 2. Future research should focus on the most appropriate mode of delivery in cases of fetal anomalies and cesarean delivery rates. Finally, the location is important: transporting a pregnant woman before delivery to a hospital that can provide a required level of care is a much safer option than transferring a neonate who requires an urgent intervention immediately after birth.

**Table 2:** Suggested management of pregnancies complicated with fetal anomalies

**After detecting fetal malformation**
**Who?** Perinatologist (specialist in maternal-fetal medicine), psychologist
The ultimate confirmation of the defect
Establishing an individual schedule of control visits, type, and frequency of monitoring
Psychological support
**After 24 weeks of gestation**
**Who? Team of specialists:** perinatologist (specialist in maternal-fetal medicine), obstetrician, neonatologist, pediatric surgeon, psychologist
The ultimate confirmation of the defect
Determining the possibility of a prenatal intervention to treat/slow down the progression of the anomaly
Determining the frequency of monitoring and recommendation about the timing, location, and mode of delivery
Psychological support
**Before labor or whenever the defect progressed**
**Who? Team of specialists:** perinatologist (specialist in maternal-fetal medicine), obstetrician, neonatologist, pediatric surgeon, psychologist
Determining the possibility of a prenatal intervention to treat/slow down the progression of the anomaly
Confirming the recommendation about the timing, location, and mode of delivery
Determining the possibility of antenatal intervention
Establishing an individual schedule of control visits
Psychological support
